# Antitumor activity and macrophage nitric oxide producing action of medicinal herb, *Crassocephalum crepidioides*

**DOI:** 10.1186/1472-6882-12-78

**Published:** 2012-06-21

**Authors:** Koh Tomimori, Shinji Nakama, Ryuichiro Kimura, Kazumi Tamaki, Chie Ishikawa, Naoki Mori

**Affiliations:** 1 Department of Microbiology and Oncology, Graduate School of Medicine, University of the Ryukyus, 207 Uehara, Nishihara, Okinawa, 903-0215, Japan; 2 Department of Psychiatry, Naha City Hospital, 2-31-1 Furujima Naha, Okinawa, 902-8511, Japan; 3 Musashino Research Institute for Immunity, 790 Nishizatozoe, Gusukube, Miyako Island, Okinawa, 906-0106, Japan; 4 Transdisciplinary Research Organization for Subtropics and Island Studies, 1 Senbaru Nishihara, Okinawa, 903-0213, Japan

## Abstract

**Background:**

*Crassocephalum crepidioides*, a plant distributed in Okinawa Islands, is known in folk medicine; however, its anticancer activity has not been investigated. The aim of this study was to determine the *in vitro* and *in vivo* antitumor activities of *C. crepidioides* on murine Sarcoma 180 (S-180) and related molecular mechanisms.

**Methods:**

The antitumor effect of *C. crepidioides* was evaluated in S-180-cell-bearing mice. Cell growth was assessed using a colorimetric assay. Nitrite and nitrate levels were measured by colorimetry. The expression levels of inducible NO synthase (iNOS) in murine RAW264.7 macrophages was assessed by reverse transcriptase-polymerase chain reaction. Activation of iNOS promoter was detected by reporter gene. Activation of nuclear factor-κB (NF-κB) was evaluated by electrophoretic mobility shift assay. The role of NF-κB signaling was analyzed using inhibitors of NF-κB and dominant-negative mutants, and Western blot analysis.

**Results:**

*C. crepidioides* extract delayed tumor growth in S-180-bearing mice. However, it did not inhibit S-180 cell growth *in vitro*. Supernatant of cultured *C. crepidioides*-stimulated RAW264.7 macrophages was cytotoxic to S-180 cells. This cytotoxicity was associated with nitric oxide (NO) production. NF-κB signaling pathway was crucial for the transcriptional activation of iNOS gene. Isochlorogenic acid, a component of *C. crepidioides*, induced NF-κB activation and iNOS expression.

**Conclusions:**

The results highlight the oncolytic and immunopotentiation properties of *C. crepidioides* mediated through NF-κB-induced release of NO from macrophages.

## Background

Plants contain several classes of phytochemicals that have antioxidative, antimutagenic and anticarcinogenic effects. The edible plant, *Crassocephalum crepidioides* S. MOORE (Japanese name; Benibanaborogiku), is wildly distributed in the Okinawa Islands and used in folk medicine for the treatment of acute hepatitis, fever and edema. Compounds with antimalarial activity and strong antimutagenicity had been isolated previously from *C. crepidioides*[1,2]. Other preparations from *C. crepidioides* have been described to have antioxidant and hepatoprotective properties [3]. However, the anticancer activity of *C. crepidioides* has not been investigated.

This is the first study that examines the antitumor effects of *C. crepidioides* extract. Although this extract is effective in inhibiting the *in vivo* growth of implanted Sarcoma-180 (S-180) cells, it did not inhibit the growth of the same cells *in vitro*. Activation of macrophages by agents such as bacterial lipopolysaccharide (LPS) stimulates their growth inhibitory effects against a wide variety of tumor cells [4]. In the present study, we report that *C. crepidioides* extract can induce the production of nitric oxide (NO), a major mediator of the tumoricidal activity of murine macrophages. In addition, serum nitrite and nitrate levels were significantly elevated in mice administered *C. crepidioides* extract compared with levels in the control group. Specifically, we characterized the mechanisms of the actions of *C. crepidioides* extract on inducible NO synthase (iNOS) promoter in murine macrophages. The antitumor efficacy of the extract was based on immunopotentiation, mediated, at least in part, by isochlorogenic acid.

## Methods

### Reagents

Fresh *C. crepidioides* was harvested in Subtropical Field Science Center of the University of the Ryukyus, Okinawa, Japan, and air-dried. Dried *C. crepidioides* (50 g) was extracted twice with 500 ml of boiling water for 30 min and the supernatant was decanted. After filtration, the combined supernatants were evaporated in vacuum and finally lyophilized to the powder. The extract obtained was used as an original extract, and dissolved with pure water when necessary. Isochlorogenic acid was purified using the procedure described previously with some modifications [3]. The extract dissolved in pure water was applied to a HP-20 (Mitsubishi Chemical, Tokyo, Japan) column eluting water and increasing amount of methanol (MeOH) to yield 70% MeOH fraction. After passing the fraction through C18 Sep-Pak cartridge (Waters, Millford, MA, USA), the final purification of the fraction was carried out by a Toyopearl HW-40 C (Tosho, Tokyo, Japan) column with 50% MeOH as an eluent. The 50% MeOH fraction contained 94% of isochlorogenic acid by absorption at 320 nm, following separation by reversed phase HPLC on C18 column (Nomura Chemical, Seto, Japan). *C. crepidioides* was dissolved in Dulbecco’s modified Eagle’s medium to a final concentration of 20 mg/ml.

Antibodies to nuclear factor-κB (NF-κB) subunits p65, p50, c-Rel and p52 were purchased from Santa Cruz Biotechnology (Santa Cruz, CA, USA). Antibody to actin was purchased from NeoMarkers (Fremont, CA, USA). Antibodies to IκBα and phospho-IκBα (Ser32 and Ser36) were obtained from Cell Signaling Technology (Beverly, MA, USA). *N*-acetyl-L-leucyl-L-leucyl-L-norleucinal (LLnL) and Bay 11-7082 were purchased from Sigma-Aldrich (St Louis, MO, USA) and Calbiochem (La Jolla, CA, USA), respectively.

### *In vivo* therapeutic effect of *C. Crepidioides*

Four-week-old female BALB/c strain athymic nu/nu mice were obtained from Ryukyu Biotec Co. (Urasoe, Japan). They were engrafted with 2 × 10^5^ S-180 cells by subcutaneous injection in the back region. Treatment was initiated on the day of cell inoculation. *C. crepidioides* was dissolved in distilled water at a concentration of 333 mg/ml, and 5 g/kg body weight of *C. crepidioides* was administered by oral gavage every day for 29 days. Tumor size was monitored once a week. All mice were sacrificed on day 28 and the tumors dissected out immediately. Tumors were fixed for paraffin embedding and tissue sectioning, and evaluated histologically using hematoxylin and eosin (H&E). This experiment was performed according to the guidelines for Animal Experimentation of the University of the Ryukyus and approved by the Animal Care and Use Committee of the same University.

### Cells

The mouse sarcoma cell line S-180 and macrophage cell line RAW264.7 were cultured in Eagle’s Minimum Essential Medium and Dulbecco’s modified Eagle’s medium supplemented with 10% heat-inactivated fetal bovine serum, respectively.

### Assays for cell growth

S-180 and RAW264.7 cells were seeded on 96-well plates and cultured for 24 h. *C. crepidioides* was added at various concentrations and incubated for 24, 48 and 72 h. In addition, the culture media were removed and replaced by supernatants from *C. crepidioides*-stimulated RAW264.7 cells. The growth of cells was evaluated by measuring the mitochondrial-dependent conversion of the water-soluble tetrazolium (WST)-8 (Nacalai Tesque, Kyoto, Japan) to a colored formazan product [5]. After 24, 48 and 72 h of culture, WST-8 (5 μl) was added to each well containing cultured cells in the last 4 h of incubation. Absorbance at 450 nm was measured using an automated microplate reader. The cell growth in untreated control cultures was considered 100%, and the growth of each treated group was compared relative to this value.

### Measurement of NO

Nitrite (NO_2_^−^), the stable end product of NO was measured in the supernatants by the colorimetric assay. Briefly, the medium was removed from individual wells and treated with Griess reagent (1% sulphanilamide and 0.1% naphtylethylene diamine dihydrochloride in 2% H_3_PO_4_) for 10 min at room temperature. The optical density of the samples was obtained using an automated microplate reader at 550 nm. A standard curve using a standard solution of NaNO_2_ in culture medium was employed to calculate the nitrite concentration. The levels of nitrite (NO_2_^−^) and nitrate (NO_3_^−^) anions derived from NO in murine sera were measured by an ENO-20 NO analyzer (EiCOM, Kyoto, Japan).

### Western blot analysis

Cells were lysed and equal amounts of protein (20 μg) were subjected to electrophoresis on sodium dodecyl sulphate-polyacrylamide gels followed by transfer onto a polyvinylidene difluoride membrane and probing with the specific antibodies. The bands were visualized with an enhanced chemiluminescence kit (Amersham Biosciences, Piscataway, NJ, USA). The reported results were obtained from at least two independent experiments with a similar pattern.

### Reverse transcriptase-polymerase chain reaction (RT-PCR)

Total cellular RNA was extracted with TRIzol (Invitrogen, Carlsbad, CA, USA). First-strand cDNA was synthesized from 1 μg total cellular RNA using an RNA-PCR kit (Takara Bio Inc., Otsu, Japan) with random primers. The primers used were 5’-TCATTGTACTCTGAGGGCTGACACA-3’ (forward) and 5’-GCCTTCAACACCAAGGTTGTCTGCA-3’ (reverse) for murine iNOS, and 5’-GTGGGGCGCCCCAGGCACCA-3’ (forward) and 5’-CTCCTTAATGTCACGCACGATTTC-3’ (reverse) or β-actin. The length of RT-PCR was 25 cycles for iNOS and 28 cycles for β-actin. The PCR products were fractionated on 2% agarose gels and visualized by ethidium bromide staining.

### Transfection and luciferase assay

The IκBαΔN- and IκBβΔN-dominant-negative mutants are IκBα and IκBβ deletion mutants lacking the N-terminal 36 and 23 amino acids, respectively [6,7]. The dominant-negative mutants of IκB kinase (IKK)α, IKKα (K44M), IKKβ, IKKβ (K44A), IKKγ, IKKγ (1-305) and NF-κB-inducing kinase (NIK), NIK (KK429/430AA) have been described previously [8,9]. pGL3 iNOS plasmid was generated by inserting the murine iNOS promoter region (−1588 to +161 bp surrounding the transcription start site) into the pGL3-basic vector (Promega, Madison, WI, USA) [10]. Three internal deletion mutants, pGL3 iNOS κB2−, pGL3 iNOS κB1− and pGL3 iNOS κB1/κB2−, were constructed by deletion of two NF-κB sites defined as the κB1 (−85 to −76) and κB2 (−971 to −962). For reporter assays, an NF-κB site-dependent luciferase vector, κB-LUC [11] was also used. RAW264.7 cells were plated and transfected with the appropriate reporter and effector plasmids using Lipofectamine reagent (Invitrogen). After 18-20 h, *C. crepidioides* was added and incubated for 6 h. The cells were lysed in reporter lysis buffer (Promega). Lysates were assayed for reporter gene activity with the dual-luciferase assay system (Promega). Luciferase activities were normalized relative to the Renilla luciferase activity from phRL-TK.

### Electrophoretic mobility shift assay (EMSA)

Nuclear extracts were obtained as described by Antalis and Godbolt [12] with modifications, and EMSA was performed as described previously [13]. The probes used were prepared by annealing the sense and antisense synthetic oligonucleotides; a κB1 site from the murine iNOS gene (5’-tcgaCCAACTGGGGACTCTCCCTTTGGGAA-3’), a κB2 site from the murine iNOS gene (5’-tcgaTGCTAGGGGGATTTTCCCTCTCTCTG-3’) and an AP-1 element of the interleukin (IL)-8 gene (5’-gatcGTGATGACTCAGGTT-3’). The above underlined sequences represent the NF-κB or AP-1 binding site. The reported results were obtained from at least two independent experiments with a similar pattern.

### Statistical analysis

Data are expressed as mean ± SD. Differences between groups were assessed for statistical significance by the Mann-Whitney’s *U*-test. A P value < 0.05 denoted the presence of a statistically significant difference.

## Results

### Effects of *C. Crepidioides* in nude mice inoculated with S-180 cells

**Figure 1:**
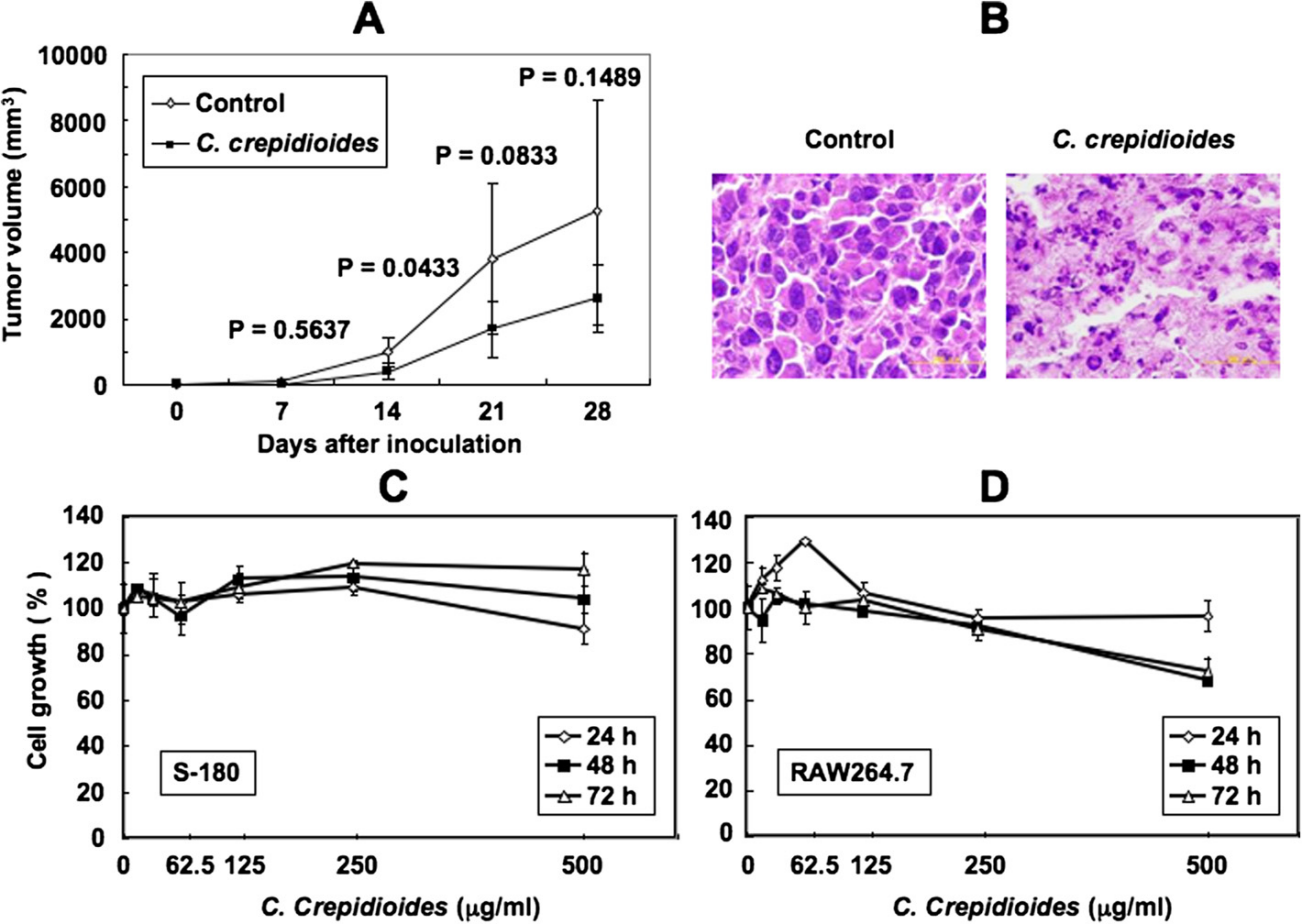
***In vivo*and*in vitro*antitumor activities of*C. crepidioides*on S-180 cells.** (**A**) Inhibition of growth of S-180 cells in nude mice. Growth of the tumors after subcutaneous inoculation of S-180 cells. Note the growth-suppressive effect of *C. crepidioides*. Data are mean ± SD of four mice in each group. The P values are the results of statistical analysis of the *C. crepidioides* and control data. (**B**) H&E-stained tissues demonstrating the pathological changes induced by treatment with *C. crepidioides*. Magnification, ×1000. (**C**) *C. crepidioides* does not have any antitumor activity on S-180 cells by itself. S-180 cells were cultured with the indicated concentrations of *C. crepidioides* extract for 24, 48 or 72 h, and cell growth was determined in triplicate cultures by WST-8 assay. (**D**) Cytotoxic effect of *C. crepidioides* on RAW264.7 cells. The cell growth was determined in triplicate cultures by WST-8 assay. Cell growth in untreated control cultures was considered 100%. Data are mean ± SD of triplicate cultures expressed as percentage of the control.

To explore the effect of *C. crepidioides* on tumor growth in nude mice transplanted with murine S-180, we treated tumor-bearing mice with 5 g/kg of *C. crepidioides* extract. The mean tumor volume was significantly lower than that of control mice after 14-day treatment (P = 0.0433, Figure 1A). However, the difference in tumor volume after 21- and 27-days treatment, compared with control, was less conspicuous. There was no significant difference in body weight gain from day 0 to day 28 between the control and *C. crepidioides*-treated groups (data not shown), and mice treated with *C. crepidioides* appeared generally healthy during the same period. On the other hand, treatment of S-180 cells with *C. crepidioides* for 29 days produced strong H&E staining for apoptosis (Figure 1B). These results suggest that *C. crepidioides* has antitumor *in vivo* effect.

### **Supernatant from*C. crepidioides*-stimulated RAW264.7 cells but not*C. crepidioides*alone causes cell growth inhibition of S-180 cells**

To study the mechanism of the antitumor effect of *C. crepidioides*, we determined the effects of *C. crepidioides* on cell growth of S-180 cells *in vitro*. Cell growth was assessed by the WST-8 assay. Incubation with *C. crepidioides* alone at concentrations up to 500 μg/ml for 72 h did not affect cell growth (Figure 1C). Activation of macrophages by agents such as bacterial LPS stimulates their growth inhibitory effects on a wide variety of tumor cells [4]. Based on this property, we investigated the effects of macrophages on S-180 cells. RAW264.7 cells were incubated with various concentrations of *C. crepidioides*. *C. crepidioides* did not have any growth inhibitory activity on RAW264.7 cells at concentrations up to 500 μg/ml for 24 h, but a mild inhibitory effect was noted at 500 μg/ml and incubation for 48 and 72 h (Figure 1D).

**Figure 2:**
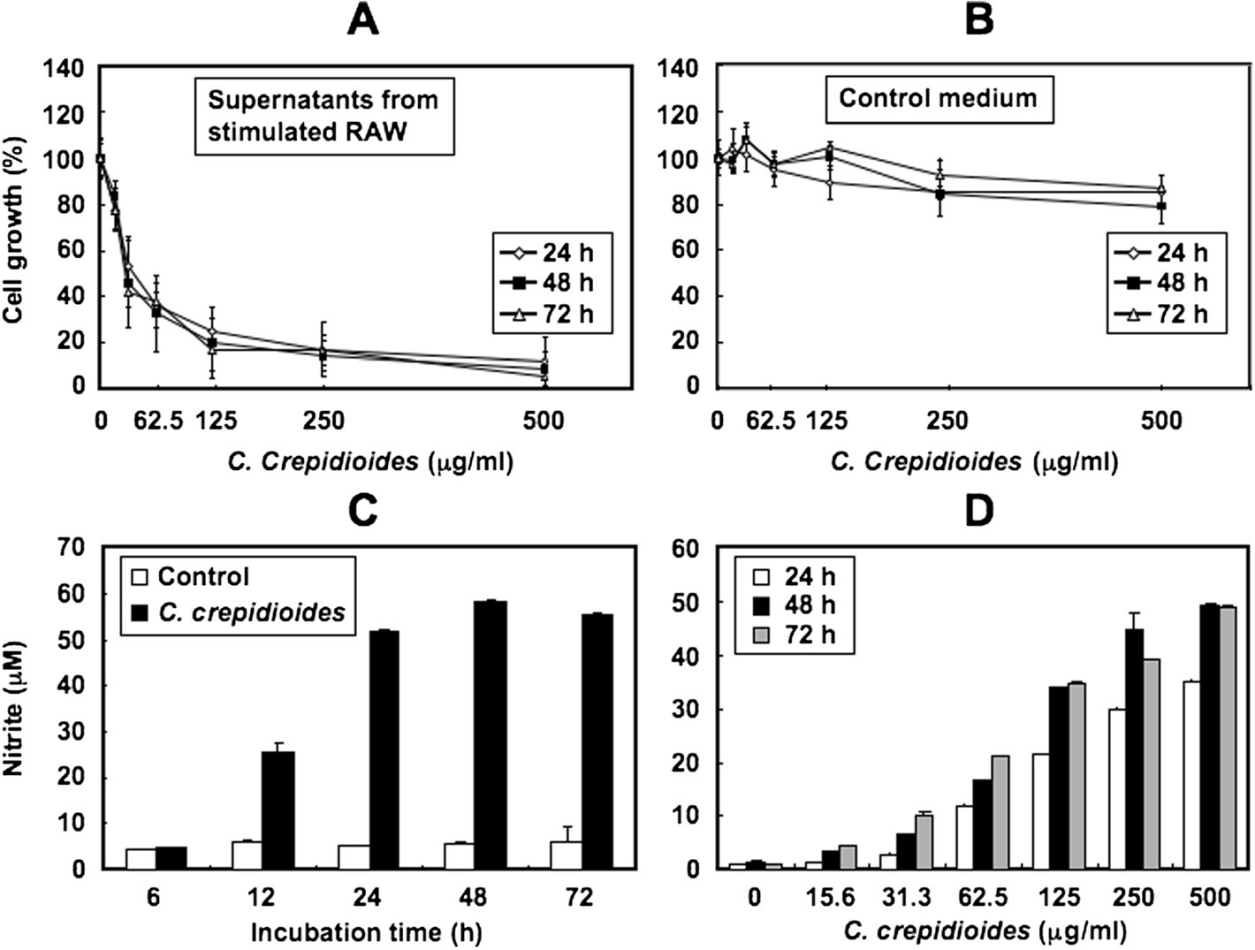
**Antitumor effect of supernatants from*C. crepidioides*-stimulated RAW264.7 cells on S-180 cells and effects of*C. crepidioides*on NO production in RAW264.7 cells.** (**A, B**) Antitumor effects of supernatants from *C. crepidioides*-stimulated RAW264.7 cells against S-180 cells, measured by WST-8 assay. RAW264.7 cells were cultured with various concentrations of *C. crepidioides* extract for 72 h. The control samples did not include RAW264.7 cells. At the end of each incubation period, the supernatant was collected. S-180 cells were precultured for 24 h. Thereafter, the culture media were removed and replaced with supernatants from *C. crepidioides*-stimulated RAW264.7 cells (**A**) or by the control medium (**B**). S-180 cells were incubated for another 24, 48 or 72 h, and cell growth was determined in triplicate cultures by WST-8 assay. Cell growth in control cultures (supernatants from unstimulated RAW264.7 cells (**A**) or control medium in the absence of *C. crepidioides* (**B**)) was considered 100%. Data are mean ± SD values expressed as percentage of the control. (**C, D**) Effects of *C. crepidioides* on NO production in RAW264.7 cells. RAW264.7 cells were incubated with or without *C. crepidioides* (500 μg/ml) (**C**) or with various concentrations of *C. crepidioides* (**D**) for the indicated time periods. NO production was determined by measuring the accumulation of nitrite in the culture medium. Data are mean ± SD of triplicate cultures.

S-180 cells were also cultured with supernatants from RAW264.7 cells that had been stimulated by various concentrations of *C. crepidioides* for 72 h. The supernatants suppressed cell growth of S-180 cells in a *C. crepidioides* dose-dependent manner (Figure 2A), while *C. crepidioides*-containing control medium had no growth inhibitory activity on S-180 cells, similar to the data shown in Figure 1C (Figure 2B).

### *C. Crepidioides* induces NO production and expression of iNOS mRNA

**Figure 3:**
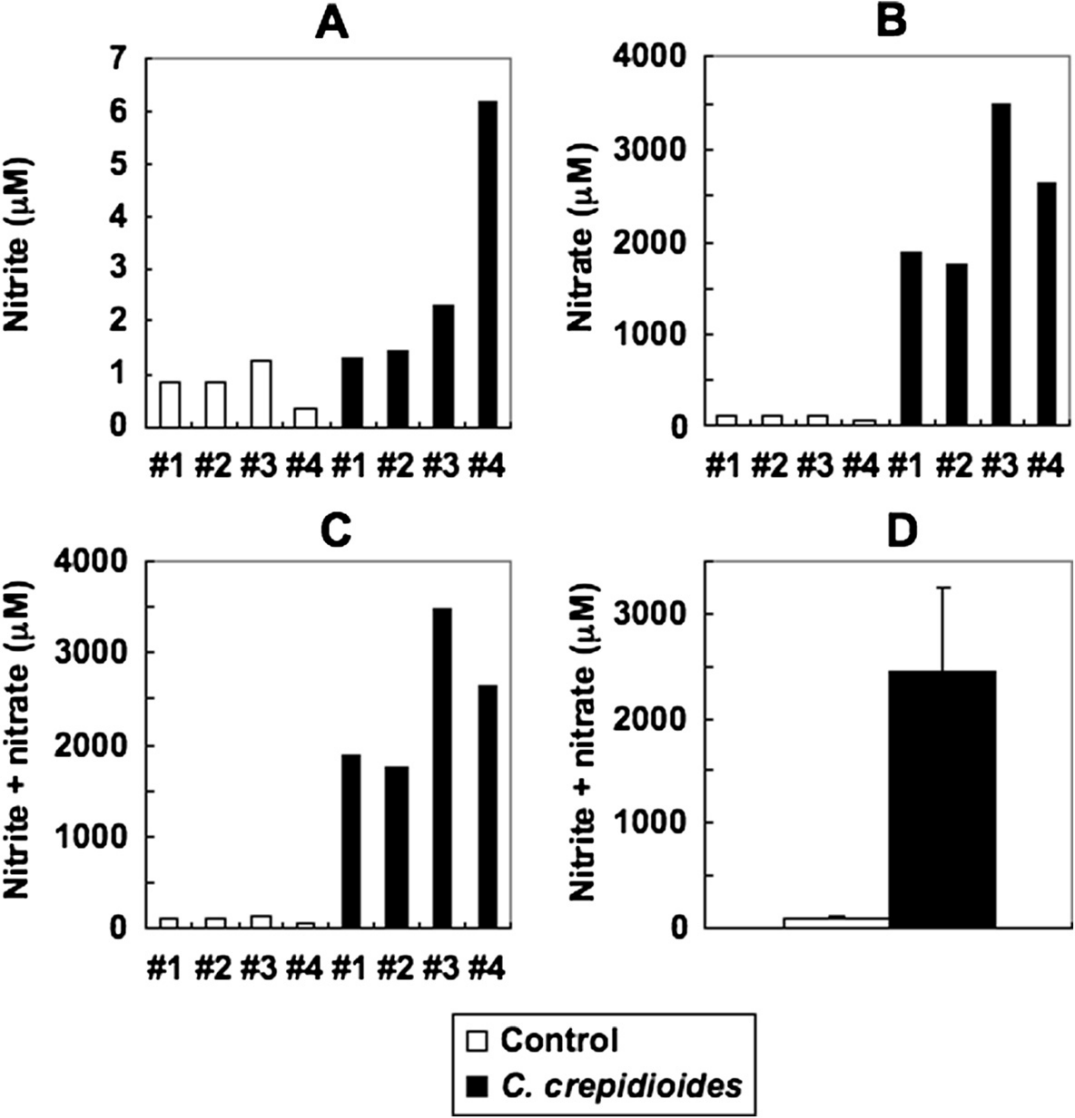
**Effect of oral administration of*C. crepidioides*(5 g/kg) on serum levels of nitrite (NO**_**2**_^**−**^**) and nitrate (NO**_**3**_^**−**^**).** (**A-C**) Serum levels of nitrite (**A**), nitrate (**B**) and nitrite plus nitrate (**C, D**) in *C. crepidioides*-treated and untreated mice at day 28. (**D**) Data are mean ± SD of four mice in each group.

Previous studies demonstrated the crucial role of NO in the tumoricidal activity of murine macrophages [14-16]. The addition of exogenous NO donor, NOR3, to the culture medium of S-180 cells inhibited cell growth (data not shown). Therefore, we examined the effects of *C. crepidioides* on NO production by RAW264.7 cells *in vitro*. NO production by stimulated RAW264.7 cells was assessed by measuring nitrite in the culture medium. *C. crepidioides* stimulated the production of nitrite from RAW264.7 cells in time- and dose-dependent manners (Figure 2C and D). In *in vivo* experiments, mice orally administered *C. crepidioides* had significantly high serum nitrite and nitrate levels compared with the control (Figure 3).

**Figure 4:**
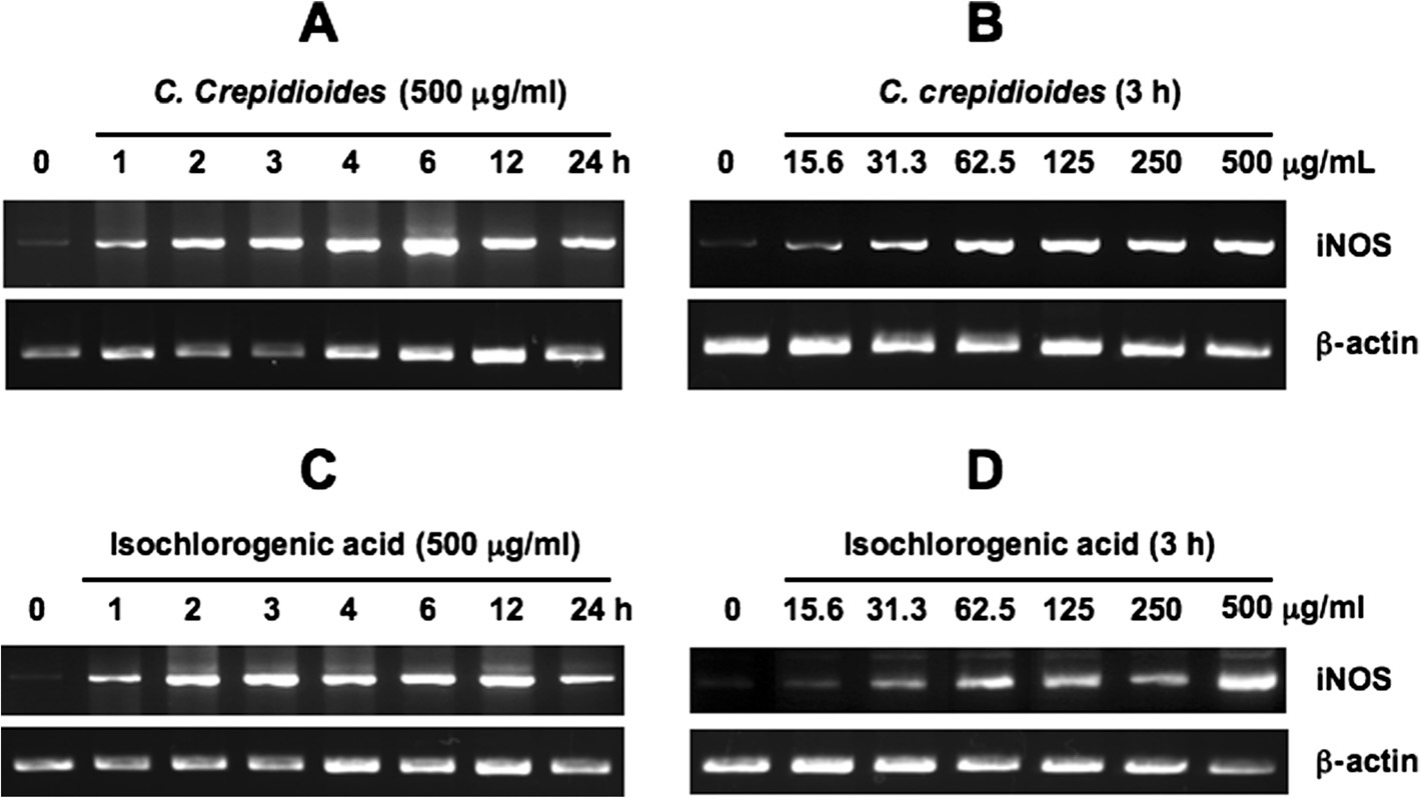
**Effects of*C. crepidioides*and isochlorogenic acid on the expression of iNOS mRNA in RAW264.7 cells.** (**A, C**) RAW264.7 cells were incubated with *C. crepidioides* (500 μg/ml) (**A**) or isochlorogenic acid (500 μg/ml) (**C**) for the indicated time periods. (**B, D**) RAW264.7 cells were cultured with various concentrations of *C. crepidioides* (**B**) or isochlorogenic acid (**D**) for 3 h. iNOS mRNA expression was detected by RT-PCR.

What is the mechanism of *C. crepidioides*-stimulated NO production? NO is synthesized by NOS-catalyzed conversion of L-arginine to L-citrulline. Whereas the activity of neuronal and endothelial NOS is mainly regulated post-translationally by cytoplasmic calcium levels or by phosphorylation by various protein kinases, iNOS is primarily regulated at the transcriptional level [17]. Next, we determined whether *C. crepidioides*-induced NO production from RAW264.7 cells was catalyzed by iNOS. The addition of 500 μg/ml *C. crepidioides* to RAW264.7 cells resulted in the expression of iNOS mRNA from 1 h after treatment (Figure 4A). In another setting, incubation with *C. crepidioides* for 3 h at 15.6 μg/ml induced iNOS mRNA expression in RAW264.7 cells (Figure 4B). The main antioxidant isolated from *C. crepidioides* is isochlorogenic acid [3]. Isochlorogenic acid stimulated iNOS expression in a manner similar to that by *C. crepidioides* (Figure 4C and D). These results suggest that the *C. crepidioides*-induced increase in NO production by RAW264.7 cells is mediated by the induction of iNOS expression, and that isochlorogenic acid seems to contribute to at least part to this augmented production of NO.

### NF-κB sites are necessary for *C. Crepidioides* inducibility of iNOS promoter

**Figure 5:**
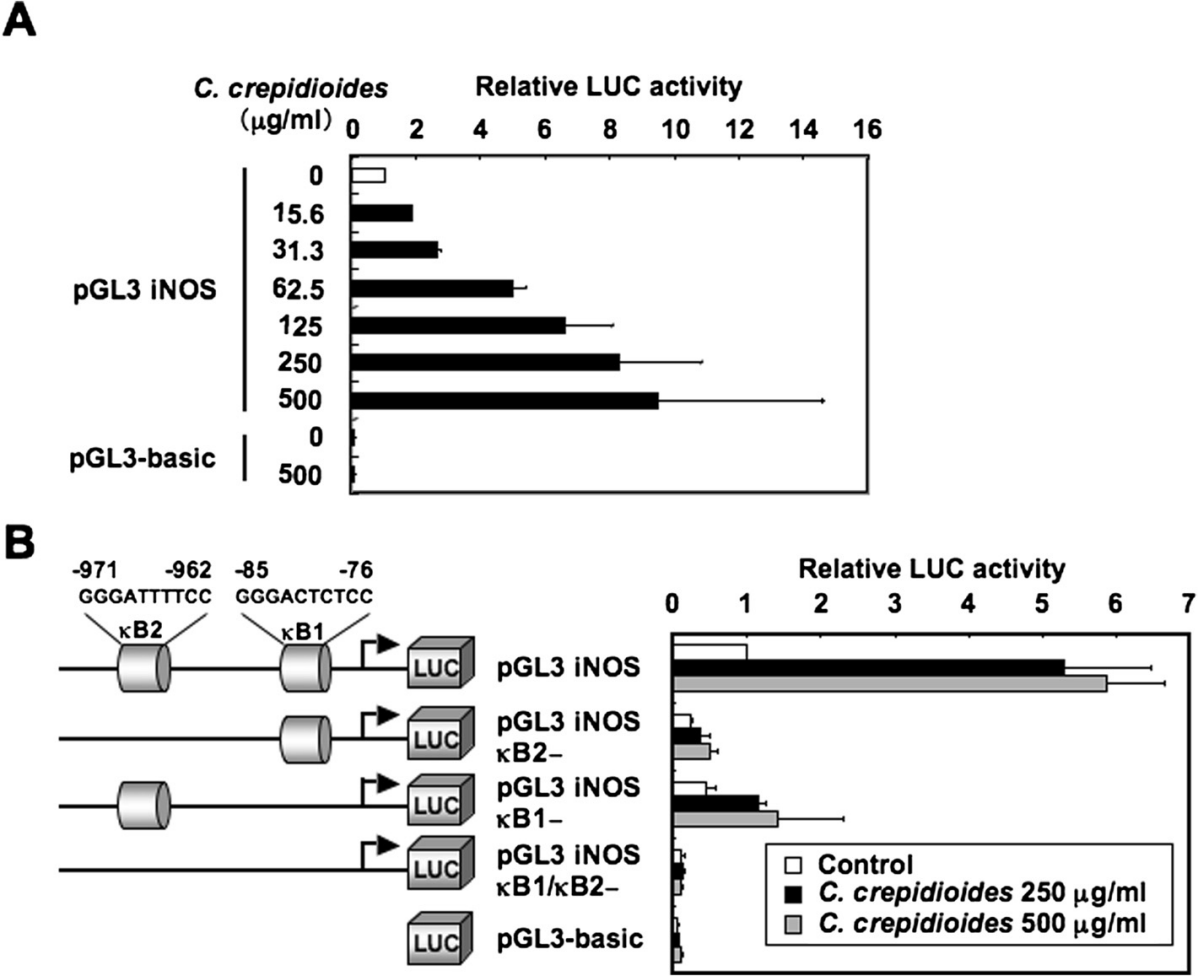
**NF-**κ**B sites in murine iNOS promoter are essential for*C. crepidioides*responsiveness.** (**A**) RAW264.7 cells were transfected with a murine iNOS promoter construct (pGL3 iNOS) or pGL3-basic, and then treated with the indicated concentrations of *C. crepidioides* for 6 h and lysed for luciferase activity analysis. All values were calculated as change (*n*-fold) in induction values relative to the basal level measured in untreated cells. (**B**) RAW264.7 cells were transfected with the indicated luciferase reporter plasmids. After transfection, the cells were left untreated or treated with *C. crepidioides* (250 and 500 μg/ml) for 6 h, and luciferase activity was measured. The activities are expressed relative to that of cells transfected with pGL3 iNOS without further treatment, which was defined as 1. Luciferase activity was normalized relative to the Renilla luciferase activity from phRL-TK. LUC, luciferase. Data are mean ± SD of three independent experiments.

To assess the effect of *C. crepidioides* on iNOS promoter activity, RAW264.7 cells were transfected with a murine iNOS-luciferase promoter/reporter construct and then incubated with various concentrations of *C. crepidioides*. The cells were lysed, and luciferase activity was measured. *C. crepidioides* increased the expression of luciferase from the iNOS promoter in a dose-dependent manner (Figure 5A). The expression of the iNOS gene in macrophages is regulated mainly at the transcriptional level, particularly by NF-κB [18-21]. The murine iNOS promoter contains two putative NF-κB binding sites, one upstream (GGGATTTTCC, −971 to −962 bp, designated NF-κB2) and one downstream (GGGACTCTCC, −85 to −76 bp, designated NF-κB1). To test the relative contribution of the NF-κB binding sites to the *C. crepidioides*-mediated activation of iNOS, we introduced a deletion into each or both sites. A single deletion of the κB2 site markedly inhibited *C. crepidioides*-mediated promoter activation, whereas a single deletion of the κB1 site resulted in moderate activation. On the other hand, double deletion completely abolished the *C. crepidioides*-mediated promoter activation (Figure 5B).

### *C. Crepidioides* induces binding of NF-κB family proteins to two NF-κB sites

**Figure 6:**
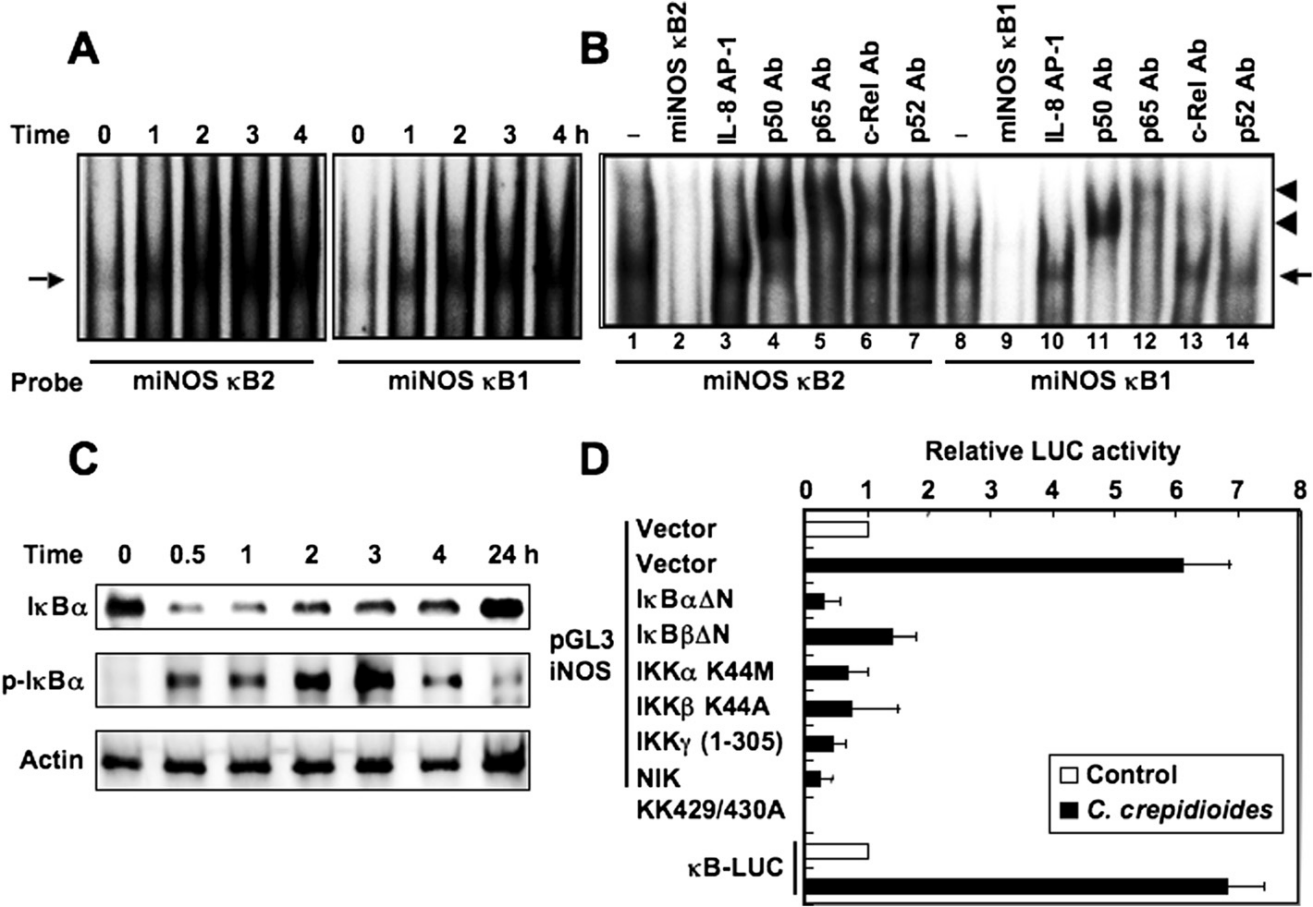
**Effects of*C. crepidioides*on signal pathway through NF-**κ**B activation.** (**A**) NF-κB1- and NF-κB2-dependent binding of nuclear proteins in RAW264.7 cells. The cells were treated with *C. crepidioides* (500 μg/ml) for the indicated time periods before preparing nuclear extracts for analysis by EMSA with probe miNOS κB1 or κB2. (**B**) Sequence specificity of NF-κB binding activity and characterization of proteins that bound to the NF-κB binding sites. Competition assays were performed with nuclear extracts from cells treated with *C. crepidioides* (500 μg/ml) for 2 h. Excess amounts of competitor (100-fold) were added (lanes 2, 3, 9 and 10). Supershift assay in the same nuclear extracts was also performed. Antibodies (Ab) were added (lanes 4-7 and 11-14). Arrows: specific complexes, arrowheads: supershifted DNA binding complexes. (**C**) *C. crepidioides* induces phosphorylation and degradation of IκBα. Cells were treated with *C. crepidioides* (500 μg/ml) for the indicated time periods and lysates were subjected to immunoblotting. (**D**) Overexpression of dominant-negative mutants inhibits *C. crepidioides*-induced activation of the iNOS promoter. Cells were transfected with pGL3 iNOS or κB-LUC and the mutant plasmids and then treated with *C. crepidioides* (500 μg/ml) for 6 h. Open bar: luciferase activity of pGL3 iNOS and empty vector or κB-LUC alone without treatment. All values were calculated as the change (*n*-fold) in induction value relative to the basal level measured in untreated cells. LUC, luciferase. Data are mean ± SD of three independent experiments.

We next characterized the nuclear proteins in *C. crepidioides*-treated RAW264.7 cells that bind to sequences from the iNOS promoter in an NF-κB-dependent manner. EMSA was performed using two probes; miNOS κB1 and κB2, oligonucleotides consisting of the NF-κB1 and NF-κB2 elements. *C. crepidioides* induced a time-dependent appearance of nuclear proteins that bound to both probes in RAW264.7 cells (Figure 6A). In both probes, the addition of excess unlabeled κB1 and κB2 oligonucleotides to the binding reaction completely abolished the formation of inducible DNA-protein complexes (Figure 6B, lanes 2 and 9). In contrast, the formation of these DNA-protein complexes was not blocked by the addition of excess of unrelated oligonucleotide AP-1 (Figure 6B, lanes 3 and 10). To identify the NF-κB family members that bind to the NF-κB motifs of the murine iNOS gene promoter, the binding reactions were preincubated with antibodies specific to p50, p65, c-Rel and p52. The anti-p50 and anti-p65 antibodies induced the supershifted bands and reduced the intensity of complexes κB1 and κB2 (Figure 6B, lanes 4, 5, 11 and 12). The c-Rel antibody also supershifted complexes κB1 and κB2 (Figure 6B, lanes 6 and 13). These results indicate that the complexes κB1 and κB2 correspond to p50/p65/c-Rel.

### NF-κB signal is essential for *C. Crepidioides*-induced iNOS expression

Does *C. crepidioides*-mediated upregulation of iNOS gene expression involve signal transduction components in NF-κB activation? Activation of NF-κB requires the phosphorylation of two conserved serine residues of NF-κB inhibitory subunit, IκBα (Ser32 and Ser36) within the N-terminal domain [22]. Phosphorylation leads to the ubiquitination and 26 S proteasome-mediated degradation of IκBα, thereby releasing NF-κB from the complex and its translocation to the nucleus and activation of various genes [22]. Next, we determined the role of IκBα phosphorylation and degradation in *C. crepidioides*-induced NF-κB translocation and activation by Western blot analysis using antibodies against phosphorylated and total IκBα. Treatment of RAW264.7 cells with *C. crepidioides* resulted in phosphorylation and degradation of IκBα within 30 min (Figure 6C).

Next, RAW264.7 cells were transfected with the luciferase reporter plasmid regulated by NF-κB elements (κB-LUC; [11]) and then incubated with *C. crepidioides* (Figure 6D). The results showed that *C. crepidioides* induced NF-κB activation. To further confirm the involvement of IκBα phosphorylation and degradation, we transfected the cells with transdominant mutant of IκBα in which two critical serine residues required for inducer-mediated phosphorylation were deleted [6]. Overexpression of mutant IκBα inhibited the *C. crepidioides*-induced iNOS promoter activation (Figure 6D), suggesting the involvement of IκBα phosphorylation and degradation in *C. crepidioides*-induced iNOS expression.

IκBβ also contains an N-terminal regulatory region required for stimulus-induced degradation, a key step in NF-κB activation [22]. Overexpression of mutant IκBβ in which two critical serine residues required for inducer-mediated phosphorylation were deleted [7], also inhibited the *C. crepidioides*-induced iNOS promoter activation (Figure 6D). These results suggest the involvement of IκBβ phosphorylation and degradation in *C. crepidioides*-induced iNOS expression.

The IKK complex consists of two catalytic subunits IKKα and IKKβ, and the regulatory subunit IKKγ [22]. In the NF-κB activation pathway, IKK complex can induce serine phosphorylation of IκBα and IκBβ [22]. Previous studies suggested the involvement of serine/threonine kinase NIK in phosphorylation and activation of the IKK complex [23]. To determine the roles of NIK, IKKα, IKKβ and IKKγ in *C. crepidioides*-induced iNOS expression in RAW264.7 cells, plasmids encoding dominant-negative mutants of NIK, IKKα, IKKβ and IKKγ were used. As shown in Figure 6D, transfection with dominant–negative mutants of NIK, IKKα, IKKβ and IKKγ significantly attenuated *C. crepidioides*-induced iNOS expression. Taken together, these findings demonstrate that *C. crepidioides* induces iNOS expression via activation of NF-κB mediated through the NIK/IKK pathway.

### Isochlorogenic acid activates NF-κB

**Figure 7:**
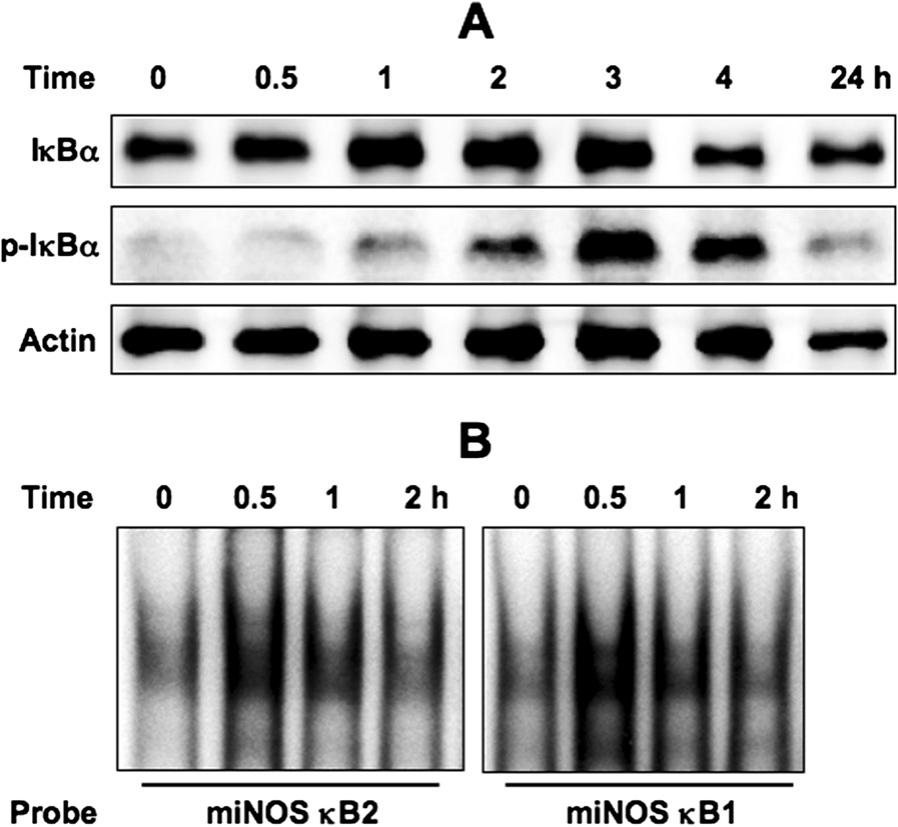
**Effects of isochlorogenic acid on NF-**κ**B activity.** (**A**) Isochlorogenic acid induces phosphorylation of IκBα. Cells were treated with isochlorogenic acid (500 μg/ml) for the indicated time periods. Lysates were subjected to immunoblotting. (**B**) NF-κB1- and NF-κB2-dependent binding of nuclear proteins in RAW264.7 cells. The cells were treated with isochlorogenic acid (500 μg/ml) for the indicated time periods before preparing nuclear extracts for analysis by EMSA with probe miNOS κB1 or κB2.

To determine the role of active compounds on *C. crepidioides*-induced iNOS expression, we examined whether isochlorogenic acid activates NF-κB. Treatment of RAW264.7 cells with isochlorogenic acid resulted in phosphorylation of IκBα (Figure 7A) and induction of nuclear proteins that bound to both miNOS κB1 and κB2 probes (Figure 7B). These results suggest that isochlorogenic acid in *C. crepidioides* seems to contribute at least in part to the induction of iNOS expression through IκBα phosphorylation and subsequent NF-κB activation.

### Effects of NF-κB inhibitors on *C. Crepidioides*-induced iNOS expression and NO production

**Figure 8:**
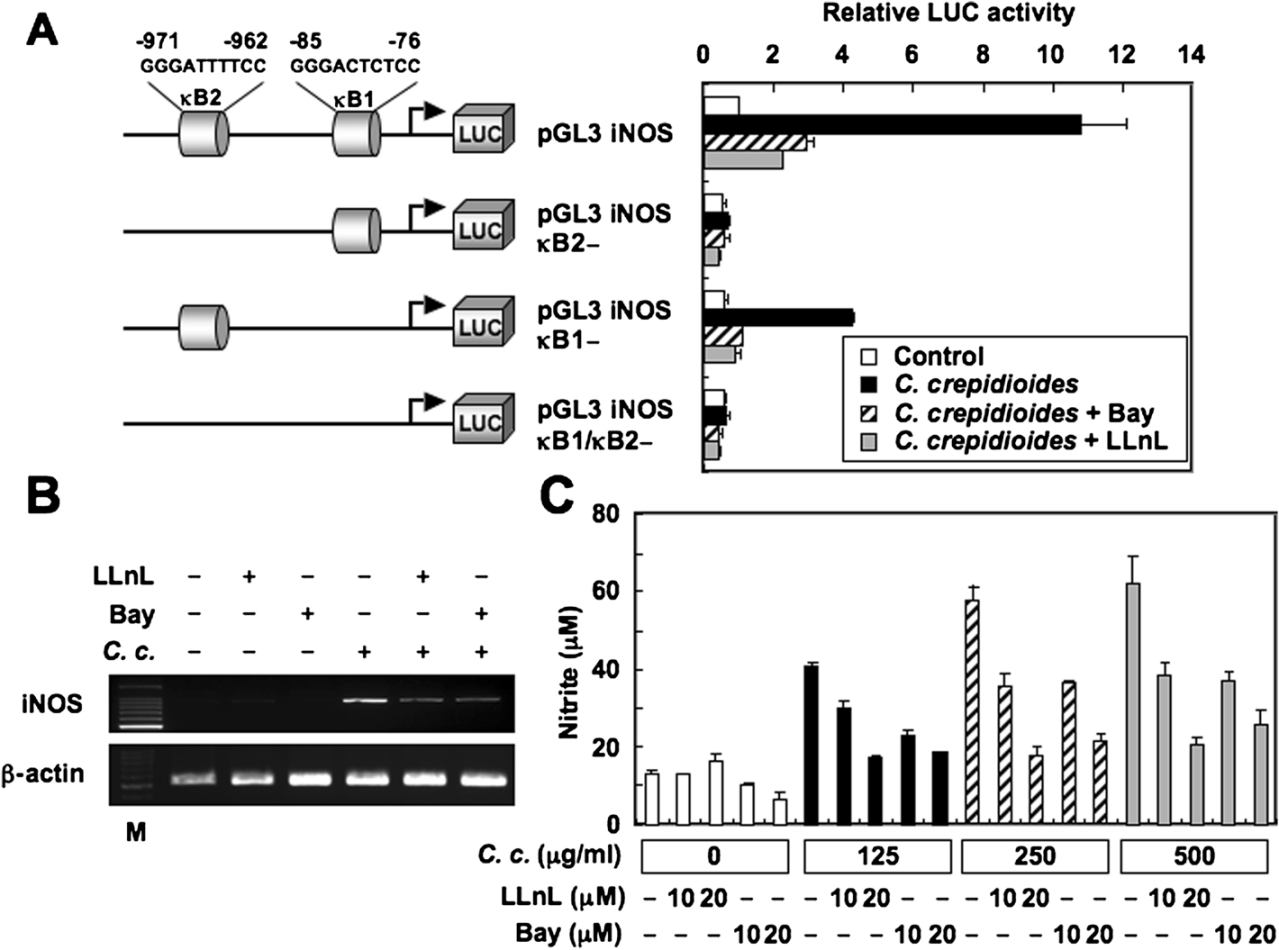
**Effects of Bay 11-7082 and LLnL on*C. crepidioides*-induced iNOS expression and NO production in RAW264.7 cells.** (**A**) iNOS reporter gene analysis. RAW264.7 cells were transfected with the indicated luciferase reporter plasmids. After transfection, the cells were incubated in a medium containing Bay 11-7082 (20 μM) or LLnL (20 μM) for 1 h and then treated with *C. crepidioides* (500 μg/ml) for 6 h. Thereafter, luciferase activities were measured. The activities are expressed relative to that of cells transfected with pGL3 iNOS without further treatment, which was defined as 1. LUC, luciferase. Data are mean ± SD of three independent experiments. (**B**) RAW264.7 cells were pretreated with Bay 11-7082 (20 μM) or LLnL (10 μM) for 1 h before stimulation with *C. crepidioides* (500 μg/ml) for another 6 h. The iNOS mRNA expression was determined by RT-PCR. (**C**) RAW264.7 cells were pretreated with the indicated concentrations of Bay 11-7082 or LLnL for 1 h before stimulation with the indicated concentrations of *C. crepidioides* for another 48 h. The nitrite content in the culture media was analyzed. Data are mean ± SD of triplicate cultures. *C. c.*, *C. crepidioides*.

Because activation of the iNOS promoter by *C. crepidioides* required the activation of NF-κB, we blocked NF-κB activation with Bay 11-7082, an inhibitor of IκBα phosphorylation [24] or LLnL, a proteasome inhibitor [25]. Bay 11-7082 and LLnL reduced *C. crepidioides*-induced iNOS promoter activity (Figure 8A). In addition, Bay 11-7082 and LLnL diminished *C. crepidioides*-induced iNOS mRNA expression and NO production in RAW264.7 cells (Figure 8B and C).

## Discussion

Alternative therapeutic tools obtained from plants to fight cancer have attracted great interest. This is the first study to report the antitumor effect of *C. crepidioides* extract in S-180 cell-bearing mice. The results demonstrated the effectiveness of *C. crepidioides* in inhibiting the growth of implanted S-180 cells, although this effect was not mirrored in the *in vitro* studies. Activated macrophages are important in host defense against tumors, including tumor cytotoxicity. NO appears to be a major mediator of macrophage tumoricidal activity [14-16]. NO secreted by activated macrophages inactivates iron-containing enzymes critical to the viability of tumor cells [26]. The present study was designed to determine whether *C. crepidioides* extract activated macrophages to the tumoricidal phenotype.

The results demonstrated that *C. crepidioides* extract alone did not inhibit S-180 cell growth *in vitro*. However, the supernatant from *C. crepidioides*-treated RAW264.7 cells inhibited S-180 cell growth. This antitumor activity was associated with NO production in activated RAW264.7 cells, and administration of *C. crepidioides* in mice induced a significant increase in serum nitrite and nitrate levels. Thus, NO seems a significant component of the pathway responsible for tumor regression. In this context, it should be emphasized that this experimental approach only allows the study of the effects mediated by soluble mediators. Other interactions involving direct contact between S-180 cells and neighboring macrophages will have additional effects on S-180 cells, modulating the proliferation and apoptosis of S-180 cells.

Another part of the present study investigated the molecular mechanism of *C. crepidioides*-induced NO production. NO formation is catalyzed by iNOS from L-arginine. Macrophage iNOS is not expressed in resting cells and differs from the constitutive neuronal and endothelial NOS [17]. Extensive studies of the transcriptional control of iNOS expression in murine macrophages have stressed the importance of binding of NF-κB [18-21], IRF-1 [27], Stat1 [28] and Oct-1 [29,30] to their recognition sequences on the iNOS promoter region for activation of iNOS transcription by interferon γ, LPS, IL-6 and Taxol. We showed here that *C. crepidioides* rapidly activated NF-κB. Moreover, we confirmed the essential role of NF-κB in *C. crepidioides*-induced iNOS promoter activity using deletion mutant forms of the two NF-κB recognition sites in the iNOS promoter, termed NF-κB1 and NF-κB2. Nuclear protein complexes that bind specifically to NF-κB1 and NF-κB2 after treatment of RAW264.7 cells with *C. crepidioides* contained p50/p65/c-Rel. The components of the NF-κB complexes formed in response to LPS consisted primarily of p50, with very low levels of p65 and c-Rel subunits [18,21]. These results support the view that LPS and *C. crepidioides* extract activate NF-κB through the same signaling pathway. Because various phytochemicals have been shown to suppress LPS-induced iNOS expression [31], we examined the effects of *C. crepidioides* extract on LPS-induced iNOS mRNA expression and NO production. However, *C. crepidioides* failed to suppress both events induced by LPS (data not shown).

Bay 11-7082 and LLnL are relatively specific inhibitors of NF-κB activation [24,25]. Both agents blocked the promoter activity of iNOS, the expression of iNOS mRNA, and the production of nitrite, indicating the likely involvement of NF-κB in the induction of not just iNOS-driven reporter constructs but also the iNOS gene itself in *C. crepidioides*-treated macrophages. We also confirmed the important role of NF-κB and the upstream target of *C. crepidioides* by showing that overexpression of dominant-negative potent inhibitors of NF-κB activation (NIK, IKKs and IκBs) inhibited *C. crepidioides*-induced activation of iNOS promoter.

Compounds with antioxidant activities have been isolated previously from *C. crepidioides*[3], including isochlorogenic acid and the flavonoids quercetin and kaempferol. However, the latter two have anti-inflammatory properties and are known to inhibit LPS-induced iNOS expression and NO production [32,33]. For this reason, isochlorogenic acid was selected in this study. Results indicated that isochlorogenic acid induces NF-κB activation and subsequent iNOS induction, and thus contributes, at least in part, to the tumoricidal effects of *C. crepidioides*.

Considered together, our results indicated that *C. crepidioides* causes regression of murine S-180 tumor and that this effect is mediated by activated macrophages through NO production. These results suggest that *C. crepidioides* is an interesting plant for the development of novel anticancer agents. However, further studies are needed to determine the effects of *C. crepidioides* extract and isochlorogenic acid on tumor growth in patients with malignant tumors.

## Conclusions

We reported in the present study the suppressive effects of *C. crepidioides* extract on tumor growth in S-180 model through the induction of NO production. Also, the extract stimulated macrophages and induced NO production via NF-κB signaling pathway. NO production may play an important role in the antitumor activity of *C. crepidioides* on S-180 cells. The results suggest that *C. crepidioides* is a potentially useful chemopreventive and chemotherapeutic agent, thus justifying further investigation of other possible beneficial biological properties.

## Competing interests

The authors declare no conflict of interest.

## Authors’ contributions

KTo designed and carried out the *in vitro* experiments. SN designed and carried out the *in vivo* experiments. RK carried out some RT-PCR experiments. KTa and CI made substantial contributions to the *in vitro* and *in vivo* experiments. NM conceived the idea, designed and coordinated the study and prepared the manuscript. All authors read and approved the final manuscript.

## Pre-publication history

The pre-publication history for this paper can be accessed here:

http://www.biomedcentral.com/1472-6882/12/78/prepub
